# Fear of war and mental health in Germany

**DOI:** 10.1007/s00127-022-02394-9

**Published:** 2022-11-29

**Authors:** A. Hajek, B. Kretzler, H. H. König

**Affiliations:** Department of Health Economics and Health Services Research, University Medical Center Hamburg-Eppendorf, Hamburg Center for Health Economics, 20246 Hamburg, Germany

**Keywords:** Fear of war, Nuclear war, War, Mental health, Depression, Anxiety

## Abstract

**Purpose:**

The objective of this study was to clarify the association between fear of war (both conventional war and nuclear war) and mental health (in terms of probable depression and probable anxiety).

**Methods:**

Data were used from the general adult population in Germany (*n* = 3091 individuals; 15th March–21st March 2022). Probable depression and probable anxiety were both quantified using validated tools (PHQ-9/GAD-7). Multiple logistic regressions were used, adjusting for several sociodemographic, lifestyle-related and health-related factors.

**Results:**

In total, 23.1% of the individuals had probable depression and 16.0% of the individuals had probable anxiety. Multiple logistic regression showed that the likelihood of probable depression was positively associated with fear of a conventional war (OR 1.25, 95% CI 1.14–1.37). Furthermore, it was associated with fear of a nuclear war (OR 1.22, 95% CI 1.12–1.33). Additionally, regressions showed that the likelihood of probable anxiety was positively associated with fear of a conventional war (OR 1.66, 95% CI 1.49–1.86). Moreover, it was associated with fear of a nuclear war (OR 1.54, 95% CI 1.39–1.71).

**Conclusions:**

Our findings stress the importance of fear of war for mental health in the general adult population in Germany. Upcoming research in this area is necessary.

## Introduction

In light of the military conflict and humanitarian crisis taking place in Eastern Europe, a turning point in history may occur.

Such events may particularly increase fear of war (both, fear of a conventional war and fear of a nuclear war) in near countries. Germany is not only such a country, but also has a very dark history of war and aggression which has shaped the attitudes of its population and its foreign policy for decades [1]. We assume that individuals in Germany may particularly fear upcoming wars.

However, thus far, little is known about the consequences of fear of war. In the 1990s, for example, Boehnke and Schwartz [2] showed that fear of war is associated with trait anxiety, but it was not associated with, among other things, negative affect or mental health (mental health subscale of the Trier Personality Inventory [3]) among University students. Some other studies from this earlier period of time exist mainly showing an association between fear of war and anxiety and depression [4–6]. Based on a convenience sample, a very recent preprint also showed an association between fear of war and mental health [7].

Given the very limited knowledge in this area, our aim was to clarify the association between fear of war (both conventional war and nuclear war) and mental health (in terms of probable depression and probable anxiety) among the general adult population in Germany. Such knowledge is of great importance because low mental health is associated with, among other things, higher healthcare costs, lower quality of life, and even higher suicidality [8–10]. This is of particular relevance since the prevalence rates are already extraordinarily high during times of the COVID-19 pandemic [11].

## Methods

### Sample

For our current study, we used data from a nationally representative online survey of people aged 18–74 in Germany (in total, *n* = 3091). The survey was conducted from March 15th to March 21st, 2022. The participants were recruited by the well-known market research firm Bilendi and respondi—an ISO 26362 certified online sample provider. Respondents were drawn from an online sample in such a way that their age group, gender, and federal state distribution corresponds to the general adult German population [12].

All participants provided informed consent. This study was approved by the Center for Psychosocial Medicine’s Local Psychological Ethics Committee at the University Medical Center Hamburg-Eppendorf (LPEK-0412).

### Outcome

The Patient Health Questionnaire-9 (PHQ-9) was used to assess probable depression. It consists of nine items [13]. It is an established and reliable screening method. A total score was computed. This score has a range of 0 to 27 with higher values reflecting more depressive symptoms. For major depressive disorder (using a PHQ-9 score of ten or higher), the sensitivity was 0.88 and the specificity was 0.88 [13]. In our study, Cronbach’s alpha was 0.89. We also used a PHQ-9 score of ten or higher as a cut-off which is in line with former recommendations [14].

The Generalized Anxiety Disorder-7 (GAD-7) [15] was used to assess probable anxiety. A total score was calculated (0–21 with higher values indicating more anxiety symptoms). Using a GAD-7 score of ten or more, the sensitivity was 0.89 and the specificity was 0.82. Cronbach’s alpha was 0.91 in our study. As recommended [15], we used a cut-off of ten or more in our current study.

### Independent variables

Fear of a conventional war and fear of a nuclear war were measured in line with prior research [2]. Individuals reported the level of fear (5-point scale from 0 = not at all worried to 4 = extremely worried) regarding “my country getting involved in a war” and “the outbreak of a nuclear war”. Prior research [2] has demonstrated that these variables are not strongly associated (*r* = 0.28) suggesting that they measure different constructs.

In light of former research [2, 4–7] and theoretical considerations, sociodemographic, lifestyle-related and health-related covariates were included as follows in regression analysis: as regards sociodemographic covariates, sex (men; women; diverse), age (years), family status (married, cohabiting with spouse; married, not cohabiting with spouse; single; divorced; widowed), having children in own household (no; yes), having a migration background (no; yes), highest educational level (Upper secondary school; Qualification for applied upper secondary school; Polytechnic Secondary School; Intermediate Secondary School; Lower Secondary School; Currently in school training/education; Without school-leaving qualification) and employment situation (full-time employed; retired; other) were included. With regard to lifestyle-related covariates, we included smoking status (yes, daily; yes, sometimes; no, not anymore; never smoker), alcohol consumption (daily; several times per week; once a week; 1–3 times per month; less often; never), and sports activities (no sports activity; less than one hour a week; regularly, 1–2 h a week; regularly, 2–4 h a week; regularly, more than 4 h a week). With regard to health-related covariates, we included chronic illnesses (absence of chronic illnesses; presence of at least one chronic illness) and self-rated health (ranging from 1 = very bad to 5 = very good).

In a robustness check, it was additionally adjusted for coronavirus anxiety (using the coronavirus anxiety scale [16–18]; score ranging from 0 to 20, with higher values indicating higher coronavirus anxiety). Cronbach’s alpha was 0.92 in our study.

### Statistical analysis

First, sample characteristics are shown stratified by probable depression as well as stratified by probable anxiety. Thereafter, multiple logistic regressions were performed to examine the association between fear of conventional war/fear of nuclear war and probable depression as well as probable anxiety, adjusting for sociodemographic, lifestyle-related and health-related factors.

The significance level was set at *p* < 0.05. Stata 16.1 (Stata Corp., College Station, Texas) was used for statistical analyses.

## Results

### Sample characteristics stratified by probable depression and probable anxiety

In the total sample, average age equaled 46.5 years (SD 15.3 years; 18–74 years) and about 49.5% were female. In Table 1, sample characteristics are shown stratified by probable depression and stratified by probable anxiety. In total, 23.1% of the individuals had probable depression and 16.0% of the individuals had probable anxiety. Moreover, 13.5% of the individuals had both probable depression and probable anxiety.

**Table 1:** Sample characteristics stratified by probable depression and probable anxiety

	Absence of probable depression (*n* = 2377; 76.9%)	Presence of probable depression (*n* = 714; 23.1%)	Absence of probable anxiety (*n* = 2595; 84.0%)	Presence of probable anxiety (*n* = 496; 16.0%)
Fear of a conventional war (0 = not at all worried to 4 = extremely worried)	2.4 (1.1)	2.7 (1.1)	2.4 (1.1)	2.9 (1.0)
Fear of a nuclear war (0 = not at all worried to 4 = extremely worried)	2.3 (1.2)	2.6 (1.2)	2.3 (1.2)	2.9 (1.1)
Gender				
Male	1270 (81.7%)	284 (18.3%)	1373 (88.4%)	181 (11.6%)
Female	1103 (72.0%)	428 (28.0%)	1217 (79.5%)	314 (20.5%)
Diverse	4 (66.7%)	2 (33.3%)	5 (83.3%)	1 (16.7%)
Age	48.1 (15.1)	41.2 (15.1)	47.6 (15.2)	40.6 (14.9)
Children in own household				
No	1650 (76.5%)	508 (23.5%)	1808 (83.8%)	350 (16.2%)
Yes	727 (77.9%)	206 (22.1%)	787 (84.4%)	146 (15.6%)
Marital status				
Single/Divorced/Widowed/Married, not cohabiting with spouse	911 (72.0%)	355 (28.0%)	1022 (80.7%)	244 (19.3%)
Married, cohabiting with spouse	1466 (80.3%)	359 (19.7%)	1573 (86.2%)	252 (13.8%)
Education				
Upper secondary school	954 (77.3%)	280 (22.7%)	1033 (83.7%)	201 (16.3%)
Qualification for applied upper secondary school	273 (76.7%)	83 (23.3%)	306 (86.0%)	50 (14.0%)
Polytechnic secondary school	152 (77.6%)	44 (22.4%)	167 (85.2%)	29 (14.8%)
Intermediate secondary school	734 (76.8%)	222 (23.2%)	798 (83.5%)	158 (16.5%)
Lower secondary school	248 (75.8%)	79 (24.2%)	273 (83.5%)	54 (16.5%)
Currently in school training/education	10 (62.5%)	6 (37.5%)	12 (75.0%)	4 (25.0%)
Without school-leaving qualification	6 (100.0%)	0 (0.0%)	6 (100.0%)	0 (0.0%)
Migration background				
No	2134 (78.4%)	587 (21.6%)	2319 (85.2%)	402 (14.8%)
Yes	243 (65.7%)	127 (34.3%)	276 (74.6%)	94 (25.4%)
Employment status				
Full-time employed	1078 (79.0%)	287 (21.0%)	1180 (86.4%)	185 (13.6%)
Retired	521 (80.7%)	125 (19.3%)	568 (87.9%)	78 (12.1%)
Other	778 (72.0%)	302 (28.0%)	847 (78.4%)	233 (21.6%)
Smoking status				
Yes, daily	528 (73.1%)	194 (26.9%)	590 (81.7%)	132 (18.3%)
Yes, sometimes	167 (70.2%)	71 (29.8%)	187 (78.6%)	51 (21.4%)
No, not anymore	743 (78.8%)	200 (21.2%)	808 (85.7%)	135 (14.3%)
Never smoker	939 (79.0%)	249 (21.0%)	1010 (85.0%)	178 (15.0%)
Alcohol consumption				
Daily	141 (70.9%)	58 (29.1%)	156 (78.4%)	43 (21.6%)
Several times a week	431 (79.2%)	113 (20.8%)	462 (84.9%)	82 (15.1%)
Once a week	374 (80.3%)	92 (19.7%)	403 (86.5%)	63 (13.5%)
1–3 times a month	429 (78.7%)	116 (21.3%)	463 (85.0%)	82 (15.0%)
Less often	562 (75.3%)	184 (24.7%)	630 (84.5%)	116 (15.5%)
Never	440 (74.5%)	151 (25.5%)	481 (81.4%)	110 (18.6%)
Sports activities				
No sports activity	606 (72.3%)	232 (27.7%)	690 (82.3%)	148 (17.7%)
Less than one hour a week	436 (75.8%)	139 (24.2%)	468 (81.4%)	107 (18.6%)
Regularly, 1–2 h a week	590 (76.5%)	181 (23.5%)	642 (83.3%)	129 (16.7%)
Regularly, 2–4 h a week	403 (82.2%)	87 (17.8%)	434 (88.6%)	56 (11.4%)
Regularly, more than 4 h a week	342 (82.0%)	75 (18.0%)	361 (86.6%)	56 (13.4%)
Chronic diseases				
Absence of at least one chronic disease	1366 (81.6%)	307 (18.4%)	1450 (86.7%)	223 (13.3%)
Presence of at least one chronic disease	1011 (71.3%)	407 (28.7%)	1145 (80.7%)	273 (19.3%)
Self-rated health (from 1 = very bad to 5 = very good)	3.7 (0.8)	3.1 (1.0)	3.7 (0.8)	3.0 (1.0)

The level of fear of a conventional war was 2.4 (SD 1.1) among individuals without probable depression and it was 2.7 (SD 1.1) among individuals with probable depression. Furthermore, the level of fear of a conventional war was 2.4 (SD 1.1) among individuals without probable anxiety and it was 2.9 (SD 1.1) among individuals with probable anxiety. Moreover, the level of fear of a nuclear war was 2.3 (SD 1.2) among individuals without probable depression and it was 2.6 (SD 1.2) among individuals with probable depression. Furthermore, the level of fear of a nuclear war was 2.3 (SD 1.2) among individuals without probable anxiety and it was 2.9 (SD 1.1) among individuals with probable anxiety. In other words, while there were small to medium differences in level of fear of a conventional war/fear of a nuclear war between individuals without probable depression and individuals with probable depression (in terms of effect size Cohen’s *d* which was in both cases about 0.3), medium differences (about *d* = 0.5 in both cases) in level of fear of a conventional war/fear of a nuclear war were identified between individuals without probable anxiety and individuals with probable anxiety. Additional sample characteristics details are given in Table 1.

### Regression analysis

Since fear of a conventional war and fear of a nuclear war were highly correlated in our study (*r* = 0.78, *p* < 0.001), we ran separate regression models where either fear of a conventional war or fear of a nuclear war was included as key independent variable. Our multiple logistic regressions are shown in Table 2 (second column: with fear of a conventional war as key independent variable and probable depression as outcome; third column: with fear of a nuclear war as key independent variable and probable depression as outcome; fourth column: with fear of a conventional war as key independent variable and probable anxiety as outcome; fifth column: with fear of a nuclear war as key independent variable and probable anxiety as outcome). It was adjusted for sociodemographic, lifestyle-related and health-related factors in all four regression models. Pseudo-*R*^2^ values ranged from 0.21 to 0.22 across the models.

**Table 2:** Fear of war and mental health

Independent variables	Probable depression	Probable depression	Probable anxiety	Probable anxiety
Fear of a conventional war	1.25***		1.66***	
	(1.14–1.37)		(1.49–1.86)	
Fear of a nuclear war		1.22***		1.54***
		(1.12–1.33)		(1.39–1.71)
Potential confounders	✓	✓	✓	✓
Pseudo-R^2^	0.22	0.22	0.21	0.21
Observations	3091	3091	3091	3091

Results of multiple logistic regressions; Odds Ratios are reported; 95% CI in parentheses; Potential confounders include sex, age, family status, having children in own household; having a migration background; highest educational level, employment situation, smoking status, alcohol consumption, sports activities, chronic illnesses and self-rated health

***p < 0.001, **p < 0.01, *p < 0.05, +*p* < 0.10

The likelihood of probable depression was positively associated with fear of a conventional war (OR 1.25, 95% CI 1.14–1.37). Furthermore, it was associated with fear of a nuclear war (OR 1.22, 95% CI 1.12–1.33).

Additionally, multiple logistic regressions showed that the likelihood of probable anxiety was positively associated with fear of a conventional war (OR 1.66, 95% CI 1.49–1.86). Moreover, it was associated with fear of a nuclear war (OR 1.54, 95% CI 1.39–1.71).

In a robustness check, the main model was extended by adding coronavirus anxiety as covariate. In this model, the associations between fear of war and the outcomes were only slightly attenuated (fear of a conventional war and probable depression, OR 1.14, 95% CI 1.03–1.26; fear of a nuclear war and probable depression, OR 1.14, 95% CI 1.04–1.24; fear of a conventional war and probable anxiety, OR 1.57, 95% CI 1.39–1.76; fear of a nuclear war and probable anxiety, OR 1.47, 95% CI 1.32–1.64).

## Discussion

Based on data from the general adult population in Germany, the purpose of this study was to investigate the association between fear of war (both conventional war and nuclear war) and mental health (in terms of probable depression and probable anxiety). In descriptive analysis, small to medium differences (in terms of effect size) were identified. Even after adjusting for various covariates, regressions showed that fear of war (both conventional war and nuclear war) was associated with a higher likelihood of both probable depression and probable anxiety.

It appears to be plausible that fear of war can contribute to a lower mental health. A higher level of fear of war can be accompanied by concerns about close friends and relatives (such as the own children) or general feelings of powerlessness in the face of a possible war [19, 20]. Fear of war can also increase death anxiety. Such factors can contribute to a lower mental health [19]. Another way to explain our current results is that fear of war contributes to pessimism (negative outlook for the future) which in turn can affect mental health [21]. Overall, our present study extends our knowledge mainly based on a few studies from the 1980s and 1990s [4–6] (which were based on specific samples) by showing a present association between fear of war and lower mental health.

Moreover, the prevalence rates reported in our study were somewhat higher compared to a previous study [22] using data from the general adult population in Germany in August/September 2021—and which was also based on the PHQ-9 and GAD-7. This former study reported a prevalence rate of probable depression of 20.0% and a prevalence rate of probable anxiety of 13.4%. These somewhat higher prevalence rates in our study may be mainly explained by the perceived double burden placed on German individuals in March 2022 (COVID-19 pandemic and particularly the military conflict and humanitarian crisis taking place in Eastern Europe).

Several strengths and limitations are worth bearing in mind. Our current study markedly extends the very limited knowledge regarding this association during these times. Data for our current study were derived from a large, representative sample (in terms of age group, sex and state). Moreover, the consequences of fear of war for mental health among adolescents and individuals in old age (75 years and over) should be further explored. Valid screening tools were used to quantify probable depression and probable anxiety.

### Limitations

While the items referring to fear of war had a high face validity, future research in this area is required to confirm our findings. Additionally, this is a cross-sectional study with its well-known limitations (e.g., regarding causality). Thus, it also appears to be plausible that depression and anxiety can contribute to fear of war. Longitudinal studies are therefore needed in upcoming research.

## Conclusion

In conclusion, our findings stress the importance of fear of war for mental health in the general adult population in Germany—and can serve as a first basis for upcoming studies. In light of the events taking place in Eastern Europe, upcoming research in this area is urgently necessary.

## Data Availability

The datasets used and analyzed during the current study are available from the corresponding author on reasonable request for all interested researchers.
